# Impact of Glycosylation and Species Origin on the Uptake and Permeation of IgGs through the Nasal Airway Mucosa

**DOI:** 10.3390/pharmaceutics12111014

**Published:** 2020-10-23

**Authors:** Simone Ladel, Frank Maigler, Johannes Flamm, Patrick Schlossbauer, Alina Handl, Rebecca Hermann, Helena Herzog, Thomas Hummel, Boris Mizaikoff, Katharina Schindowski

**Affiliations:** 1 Institute of Applied Biotechnology, University of Applied Science Biberach, Hubertus-Liebrecht Straße 35, 88400 Biberach, Germany; ladel@hochschule-bc.de (S.L.); maigler@hochschule-bc.de (F.M.); flamm@hochschule-bc.de (J.F.); schlossbauer@hochschule-bc.de (P.S.); handl@hochschule-bc.de (A.H.); rebecca.hermann96@gmail.com (R.H.); helena.herzog@uni-ulm.de (H.H.); 2 Faculty of Natural Science, University of Ulm, Albert-Einstein-Allee 11, 89081 Ulm, Germany; 3 Smell & Taste Clinic, Department of Otorhinolaryngology, TU Dresden, Fetscherstraße 74, 01307 Dresden, Germany; thummel@msx.tu-dresden.de; 4 Institute of Analytical and Bioanalytical Chemistry, University of Ulm, Albert-Einstein-Allee 11, 89081 Ulm, Germany; boris.mizaikoff@uni-ulm.de

**Keywords:** IgG permeation, barrier model, nose-to-brain, primary cells, RPMI 2650, olfactory epithelium, respiratory epithelium, drug delivery, blood-brain barrier

## Abstract

Although we have recently reported the involvement of neonatal Fc receptor (FcRn) in intranasal transport, the transport mechanisms are far from being elucidated. Ex vivo porcine olfactory tissue, primary cells from porcine olfactory epithelium (OEPC) and the human cell line RPMI 2650 were used to evaluate the permeation of porcine and human IgG antibodies through the nasal mucosa. IgGs were used in their wild type and deglycosylated form to investigate the impact of glycosylation. Further, the expression of FcRn and Fc-gamma receptor (FCGR) and their interaction with IgG were analyzed. Comparable permeation rates for human and porcine IgG were observed in OEPC, which display the highest expression of FcRn. Only traces of porcine IgGs could be recovered at the basolateral compartment in ex vivo olfactory tissue, while human IgGs reached far higher levels. Deglycosylated human IgG showed significantly higher permeation in comparison to the wild type in RPMI 2650 and OEPC, but insignificantly elevated in the ex vivo model. An immunoprecipitation with porcine primary cells and tissue identified FCGR2 as a potential interaction partner in the nasal mucosa. Glycosylation sensitive receptors appear to be involved in the uptake, transport, but also degradation of therapeutic IgGs in the airway epithelial layer.

## 1. Introduction

In the last 30 years, the rise in the importance of therapeutic immunoglobulin G (IgG) has been exceptional. Two Nobel prizes have been awarded to scientists for antibody-related discoveries so far, over 80 antibodies have entered clinical trials and the approval success rates have been near one in four [1,2]. The use of therapeutic IgGs in indications such as oncology, autoimmune diseases and inflammatory diseases represented revolutionary innovations in the treatment of chronic and acute conditions. However, up to now, the use of IgGs in central nervous system (CNS)-related diseases is severely hampered due to the low blood–brain barrier (BBB) permeability and the poor brain permeability in general. The four distinct and highly restrictive barrier structures, the BBB, the blood–CSF (cerebrospinal fluid) barrier, the meningeal barrier and the ventricular barrier, are the most limiting factors in successful IgG-based therapy of CNS-related diseases. Amongst them, the BBB is the most relevant as it provides access to the entire brain [3]. There are several mostly experimental strategies to overcome this barrier. One strategy is to disrupt the tight junctions in the BBB to allow paracellular passage of molecules [4]. Furthermore, mannitol, an osmotic agent that leads to a shrinkage of the brain endothelial cells or focused ultrasound can be used to increase the permeability to the brain [5,6]. However, the unspecific disruption of the BBB harbors the risk of exposing the brain to potentially harmful bloodborne substances [3]. Besides the unspecific trafficking strategies to the brain, also selective approaches were performed using receptors, such as the transferrin receptor, that are known to undergo transcytosis in the brain endothelial cells [3,7,8,9]. Alongside the strategies described, there are also ideas to circumvent the BBB, including the concept of nose-to-brain (N2B) drug delivery. In theory, N2B is most likely mediated along the olfactory or the trigeminal nerve pathways that are located in the neuroepithelium of the nasal cavity or by paracellular transport [10,11,12]. In a former IgG permeation study through ex vivo porcine olfactory mucosa explants, IgGs were found in olfactory epithelial cells, glands and in neuronal fiber tracts [13]. Hereby, differences between the permeation of allogenic porcine IgGs and xenogenic human IgGs have been shown. It was assumed that the neonatal Fc receptor (FcRn), a specialized IgG transporter that is expressed mainly in endothelial and epithelial cells as well as monocytes, is involved in IgG uptake and trafficking in the neuroepithelium. Accordingly, Stirling et al. showed higher uptake of human IgG compared to porcine IgG in porcine kidney cells expressing the FcRn [14]. In accordance, our previous data may indicate the faster penetration of human IgG through porcine olfactory mucosa explants compared to the permeation of porcine IgG in the same set-up [13].

In general, several studies show the involvement of FcRn in IgG transport in different tissues and cell types [15,16,17,18,19]. Thus, FcRn was also found to be expressed in the BBB. The role of FcRn in the BBB appears to be mainly as an efflux transporter; however, some studies suggest that FcRn may also take part in IgG distribution within the brain [20,21,22]. There are many controversial studies concerning the influence of these Fc receptors in IgG distribution in the brain. Recently, Ruano-Salguero and Lee showed in an in vitro BBB model that antibody transcytosis across brain endothelial-like cells is not dependent on FcRn transport [23]. However, they also highlighted the need for clarifying the transcytosis mechanism in general. Nevertheless, the function of FcRn as IgG transporter in the mucosal epithelium was proven in several studies focusing on gut or lung mucosa [16,24]. As the nasal mucosa displays the first barrier to overcome for intranasally administered therapeutic IgGs, it is of great interest to evaluate if FcRn function is similar to other reported mucosal sites or if the function of FcRn in the neuroepithelium is comparable to neuronal tissue. Other candidates that are discussed to have potential IgG transport functions and could be involved in mucosal IgG trafficking are Fc gamma receptors (FCGRs), particularly FCGR2b [25,26]. Again, the influence of these FCGRs on the transport of antibodies has not been clarified yet. These receptors are mainly known to mediate immunological effector functions and are highly dependent on IgG glycosylation [27,28]. Several studies show altered interaction of FCGR with deglycosylated IgGs. EndoS deglycosylation of IgG1 leads to decreased binding to human FCGR2a/b and FCGR3a, whereas binding to FCGR1 is not affected. Interestingly, reduced fucosylation leads to increased FCGR3a binding, as shown by Cambay et al. [27,29,30,31,32].

The present study focuses on the difference in permeation rates of different IgGs (allogenic porcine IgG and xenogenic human IgG, both glycosylated and deglycosylated) through different models for nasal drug delivery: ex vivo specimens of porcine olfactory mucosa, primary epithelial cells derived from porcine olfactory mucosa and the human cell line derived from a squamous nasal epithelial cancer, RPMI 2650. Furthermore, the influence of IgG glycosylation on permeation was investigated by comparing the normally glycosylated (wild type, WT) IgG and enzymatically deglycosylated (DG) IgG. Because of the lack of commercially available monoclonal porcine IgGs, a biosimilar of a well characterized human IgG against vascular endothelial growth factor (VEGF), named hIgG in the following, was used for the deglycosylation study as a defined glycopattern was preferable. Porcine mucosa was used for modeling the human nasal mucosa as its high similarity was described before in other studies and the cross-species transport of human IgG by the porcine FcRn has been already shown by Stirling et al. [13,14,33,34,35]. In addition, the permeation experiments were also performed in porcine olfactory epithelial primary cells (OEPC) and in the human cell line RPMI 2650 (ACC 287, DSMZ, Braunschweig, Germany) [36,37,38,39]. The most important factor limiting the nasal absorption of high molecular weight molecules is the low epithelial membrane permeability and the mucociliary clearance [40]. Both factors were included in the cellular OEPC model, displaying the first barrier for N2B. In the ex vivo model, more complex factors interfere with IgG permeation, such as the interaction with the local immune system and the uptake in neuronal fibers, which makes it more similar to the in vivo and, hence, clinical situation.

## 2. Materials and Methods

### 2.1. IgGs Used in This Study

To evaluate the permeation kinetics of a human IgG, hIgG was produced in an in-house project according to standard procedures and purified with Protein A MabSelect SuRe™ resin (GE Healthcare, Solingen, Germany) on an ÄKTA Purifier system (GE Healthcare, Solingen, Germany). The antibody was eluted with 50 mM sodium acetate buffer pH 4.0. The concentration was determined at 280 nm using a NanoDrop™ (Thermo Fisher Scientific, Dreieich, Germany) spectrophotometer applying the extinction coefficient 1.54 L/(mol × cm).

Porcine serum IgGs were purchased from Sigma-Aldrich (Taufkirchen, Germany).

### 2.2. IgG Deglycosylation

IgGs were deglycosylated by the enzyme Endo S (NEB, Ipswich, UK) according to the manufacturer’s protocol. Briefly, 500 µg IgG was incubated with 340 U Endo S for 2 h at 37 °C. Endo S was separated from the IgG by ultrafiltration (100 kDa MWCO, Satorius, Göttingen, Germany). The deglycosylation was confirmed by hydrophobic interaction liquid vhromatography (HILIC, see Supplementary Figure S1). Hereby, the exact glycopattern was determined by analyzing the cut-off Fc-glyco-residues. The identification of the glycosylation pattern is both a quality control for the IgG itself, as the glycopattern highly depends on the production process, and a verification of a successful deglycosylation process as, otherwise, no glycan-residues would be found by HILIC.

### 2.3. Tissue Preparation and Ex Vivo Permeation

The mucosa explants were harvested and placed upside-down in a side-by-side set-up, as described before [13]. In brief, tissue specimens (2 cm^2^) were excised from the *concha nasalis media* (*c.n. media*) of slaughterhouse pigs (postmortem delay < 1.5 h). To simulate the upside-down conditions at the olfactory region, the mucosa specimens were placed into a self-made side-by-side cell system consisting of two 1.5 mL microreaction tubes. The upper compartment was filled with 0.26 mL DMEM/F12 (Gibco^®^ Invitrogen, Darmstadt, Germany) supplemented with 20 U penicillin, 20 µg streptomycin (PenStrep (U), AppliChem, Darmstadt, Germany) and 300 I.U./mg gentamycinsulfate (≥590 I.U./mg, Carl Roth, Karlsruhe, Germany). Then, 30 µL of 1.5 mg/mL IgG solution in PBS pH 6.5 was applied onto the epithelial layer (lower compartment). The system was incubated at 35 °C and >90% humidity for 5 h. As negative/vehicle control PBS without IgG was used. After permeation, the mucosa explants were directly fixed in 4% paraformaldehyde for 2 h and cryoconserved in 30% sucrose overnight. Then, 14 µm slices were prepared in a cryostat at −25 °C (HM525 NX, Thermo Fisher Scientific, Dreieich, Germany) and mounted on Superfrost^®^Plus Micro Slides (VWR International GmbH, Darmstadt, Germany). To verify tissue integrity, adjacent or following slides from each sample were hematoxylin–eosin stained (HE; Gil III, Merck Millipore, Darmstadt, Germany, data not shown) as described previously [13]. Ex vivo permeation was performed in four independent experiments at least. Samples with damaged epithelial layers were excluded from the study as described [13].

### 2.4. Cell Culture

#### 2.4.1. Primary Cells

Porcine olfactory epithelial primary cells (OEPC) were prepared and cultured as described recently [36]. Briefly, cells were harvested from mucosal explants originating from the dorsal part of the *concha nasalis dorsalis* (*c.n. dorsalis*) and the *c.n. media*. The cells were disinfected with Octenisept (Schülke, Noederstedt, Germany), washed and isolated by pronase digestion (1.4 mg/mL in EBSS (Gibco^®^ Invitrogen, Darmstadt, Germany), 20 U penicillin, 20 µg streptomycin (PenStrep (U), AppliChem, Darmstadt, Germany), 300 I.U./mg gentamycinsulfate (≥590 I.U./mg, Carl Roth, Karlsruhe, Germany) for 1 h at 37 °C) and agitation. The suspension was carefully removed from the remaining tissue and centrifuged at 700 rpm for 3 min. The sedimented cells were taken up in appropriate volumes of primary cell culture adhesion medium (DMEM:F12 (1:1), 20% fetal bovine serum (FBS) 2 mM Gln, 1% non-essential amino acids (NEAA), 20 U penicillin, 20 µg streptomycin, 300 I.U./mg gentamycinsulfate) and seeded in T75 flasks coated with 0.05 mg/mL rat tail collagen solution (Primacyte, Schwerin, Germany). Cells were cultivated at 37 °C, 5% CO_2_ and 95% rH. The medium was changed to primary cell culture medium (DMEM:F12 (1:1), 10% FBS, 2 mM Gln, 1% NEAA, 20 U penicillin, 20 µg streptomycin, 300 I.U./mg gentamycinsulfate) after 6 h. Fibroblasts were removed by regular Trypsin/EDTA incubation for 4 min at 37 °C.

#### 2.4.2. RPMI 2650

RPMI 2650 cells were cultivated in MEM medium (BioWest, Nuaillé, France) containing 10% FBS, 2 mM Gln and 10U penicillin, 10 µg streptomycin at 37 °C, 5% CO_2_. 95% rH. Cells were split on a regular basis at 80–90% confluency by trypsination (Trypsin/EDTA, Biochrom, Stockelsdorf, Germany).

#### 2.4.3. Air–Liquid Interface (ALI) Cell Culture

**Figure 1:**
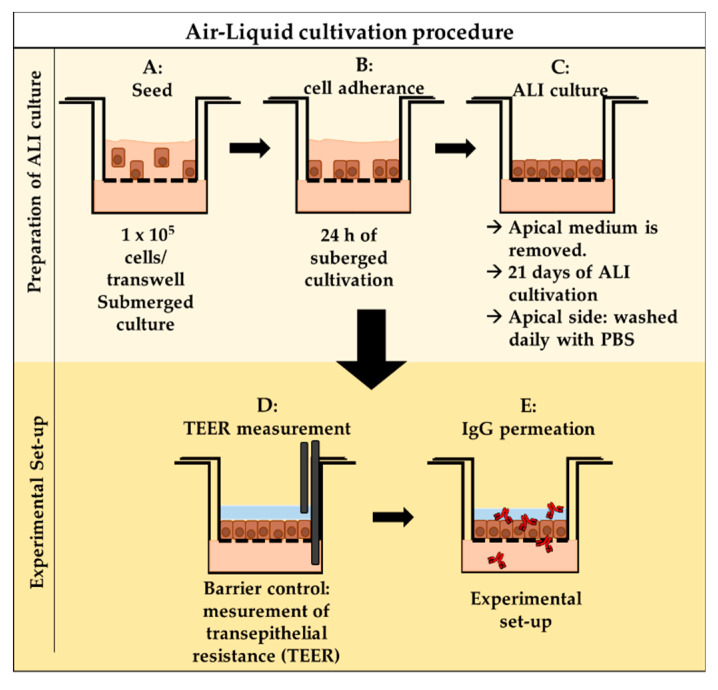
Workflow for air–liquid interface (ALI)-based in vitro permeation experiments. (**A**) Cells were seeded in a concentration of 1 × 10^5^ cells/transwell. (**B**) Cell were incubated for 24 h to adhere to the inserts. (**C**) After 24 h under submerged conditions, the medium from the apical side was removed to achieve ALI conditions. The cells were washed once daily with PBS to avoid the medium draining from the bottom to the apical surface. Cells were differentiated for 21 days at ALI conditions for an optimal and tight barrier. (**D**) Barrier integrity was confirmed by transepithelial resistance (TEER) measurement. (**E**) Experimental set-up of IgG permeation.

To expose airway epithelial cells with their apical sides to the air, while their basolateral sides still have contact with the medium, the cells were cultured on cell culture inserts at the air–liquid interface (ALI). This procedure mimics the conditions in the airways. For permeation experiments, primary cells and RPMI 2650 cells were seeded in collagen-coated (0.05 mg/mL rat tail collagen solution) cell culture inserts (ThinCert^TM^, Greiner Bio-one, Frickenhausen, Germany) with a density of 1 × 10^5^ cells (diluted in culture medium), and 260 µL cultivation medium was applied in the basolateral compartment. The apical volume was removed after 24 h of cultivation under submerged conditions. For an additional 21 days, the cells were cultivated under ALI conditions. To remove diffused medium (and prevent fibroblast outgrowth in the primary cell culture), the apical side of the ALI cultures was washed every other day with 200 µL pre-warmed PBS. After 24 h, the cells were adherent and were not removed by the washing steps. In addition, the basolateral medium was also replenished every other day. The ALI procedure is visualized in Figure 1A–C.

### 2.5. In Vitro Permeation

#### 2.5.1. TEER Measurement

The trans-epithelial electrical resistance (TEER) measurement was used to confirm cell layer integrity (Figure 1D). Cell culture inserts were apically filled with 350 µL MEM without phenol red (Gibco^®^, Invitrogen, Schwerte, Germany) and the abluminal/basolateral compartment filled with 500 µL MEM. With incubation for 20 min at 37 °C and 15 min at room temperature (RT), the cells were allowed to equilibrate to the new conditions. The TEER values were determined in triplicate using an EVOM epithelial voltohmmeter and chopstick electrodes (World Precision Instruments, Friedberg, Germany). The raw data were processed by subtracting the blank (inserts without cells) and by multiplying with the area of the insert’s membrane (0.336 cm^2^). The cut-off values for OEPC and RPMI 2650 confluent cell layers were determined as described previously [36].

#### 2.5.2. In Vitro Permeation—Experimental Procedure

To carry out the permeation experiments, the medium was changed to 260 µL cultivation medium without FBS to avoid potential interference of traces of bovine IgGs within the FBS. To analyze IgG permeation, 100 µL of 0.5 mg/mL IgG in PBS was applied at the apical surface of the cell layer (Figure 1E). The experiments were executed under normal cell culture conditions at 37 °C, 5% CO_2_, 95% rH. Permeation was performed for 48 h. For sampling, a volume of 20 µL was taken from the basolateral compartment at 0.5, 2, 4, 8, 12, 24 and 48 h. Then, 20 µL fresh medium was added to keep the basolateral volume constant. The samples were analyzed via enzyme-linked immunosorbent assay (ELISA). The remaining cell culture inserts were fixed in 4% paraformaldehyde for 10 min, cryoconserved in 30% sucrose overnight and stored at 4 °C until sectioning.

The samples were analyzed by ELISA. For the quantitative analysis of hIgG, a sandwich set-up was used. High-binding 96-well plates (Microlon, Greiner Bio-one, Frickenhausen, Germany) were coated with 0.05 µg/mL of recombinant VEGF (Sino Biologicals, Beijing, China) using a 0.1 M bicarbonate buffer pH 9.6 overnight at 4 °C. Plates were washed four times with PBS supplemented with 0.3% Tween (PBST) for 1 min, followed by a blocking step with 1% bovine serum albumin (BSA) in PBST and incubated for at least 2 h at 37 °C or overnight at 4 °C. Samples were diluted in coating buffer, added to the wells and incubated for 45 min at 37 °C. Plates were washed again four times as described before. The detection antibody anti-human κ (from goat) conjugated with horseradish peroxidase (HRP; Table 1) was diluted 1:4000 in coating buffer, added to the wells and incubated for 30 min at 37 °C. After extensive washing with PBST, TMB chromogenic substrate (Thermo Fisher/Pierce^TM^, Dreieich, Germany) was used for detection according to the manufacturer’s protocol.

The quantitative analysis of porcine IgGs was also carried out using the sandwich ELISA (company, city, country) used for hIgG despite the coating protein and the detection antibody. Here, AffiniPure goat anti-swine IgG (H + L) (Table 1) was coated with a concentration of 1µg/mL. For detection, the goat anti-swine Fc conjugated with HRP (EMD Millipore, Darmstadt, Germany) was used in a 1:50,000 dilution. Absorbance was read at 450 nm using the SpectraMax M Series (molecular devices, San Jose, CA, USA).

### 2.6. Immunofluorescence Staining of Tissue Explants and Cell Culture Insert Membranes

The immunofluorescence (IF) staining was performed as described previously [13,36]. Briefly, sections/membranes were washed three times with PBS pH 7.4 for 5 min, followed by blocking with 4% BSA, 0.5% saponin and 10% normal goat serum in PBS pH 7.4 overnight. The primary antibodies against FcRn (Pirbright Institute, Woking, UK) and against FCGR2/CD32 (Novus Biologicals/Bio-Techne GmbH, Wiesbaden, Germany) were diluted 1:100 in PBS pH 7.4 containing 4% BSA and 0.5% saponin and incubated for 24 to 48 h at 4 °C. Afterwards, the sections/membranes were washed again three times and incubated with the corresponding secondary antibody (Table 1, 1:500 diluted in PBS pH 7.4) for 2 h. Subsequently, three additional washing steps were performed. Nuclei were stained via DAPI (20 µg/mL in PBS, Thermo Fisher, Dreieich, Germany) for 10 min and washed in PBS pH 7.4. After three additional washing steps, slides were mounted with Fluoromount G mounting medium (Sigma-Aldrich, Taufkirchen, Germany).

### 2.7. Western Blot Analysis

The Western blot was performed as described before [36]. Briefly, the cell and tissue lysates with equal weight or cell count were extracted by using ice-cold RIPA cell lysis (10 mM Tris-Cl, pH 8.0; 1 mM EDTA, 0.5 mM EGTA, 1% Triton X-100; 0.1% sodium deoxycholate, 0.1% SDS, 140 mM NaCl; protease inhibitor mix (Thermo Fisher Scientific, Dreieich, Germany) and ultrasound (Hielscher, Stuttgart, Germany)). Equal volumes of homogenized tissue were loaded, separated in a 12.5% SDS PAGE and blotted onto a nitrocellulose membrane (Carl Roth, Karlsruhe, Germany). The membrane was blocked with 5% skimmed milk powder (TSI GmbH, Zeven, Germany) in PBS-0.1% Tween20, pH 7.4 for 1 h at room temperature (RT). Primary antibodies against FcRn [14]; for human: Sigma Aldrich, Taufkirchen, Germany) and against β-Actin (Sigma Aldrich, Taufkirchen, Germany) were diluted 1:5000 in blocking buffer and incubated overnight at 4 °C. Secondary antibodies were diluted 1:5000 (Anti-human IgG-HRP, SouthernBiotech, Birmingham, AL, USA), 1: 50000 (Anti-rabbit IgG-HRP, Jackson Immuno Research, Ely, UK) and 1:4000 (Anti-murine IgG–HRP, Sigma Aldrich, Taufkirchen, Germany). For signal detection, the chemiluminescence substrate Immobilon^®^ (Merck Millipore, Darmstadt, Germany) was used according to the manufacturer’s instructions.

### 2.8. Immunoprecipitation

Immunoprecipitation was performed to identify interaction partners of IgG in cells and the tissue. Tissue and cell lysates were prepared as described in Section 2.7 with 1% Triton X-100, 20 mM Tris, 150 mM NaCl, pH 6.5 as lysis buffer. Pierce^TM^ Protease and Phosphatase Inhibitor (Thermo Scientific, Dreieich, Germany) was freshly added 1:1000 to the working solution. Lysates were centrifuged in a microcentrifuge (Eppendorf, Hamburg, Germany) at maximum speed for 15 min at 4 °C. Then, 500 µL of the supernatant was added to 50 µg of the capture antibody and incubated by overhead agitation for 1 h at 4 °C. Then, 20 µL of Protein A agarose beads (Cell Signaling Technology, Frankfurt, Germany) was added and incubated under agitation overnight at 4 °C. Beads were washed four times with 500 µL ice-cold lysis buffer. The pellet was resuspended with 20 µL 3× SDS sample buffer, mixed by vortexing and centrifuged for 30 s at maximum speed. Proteins were eluted from the beads by boiling for 5 min at 95 °C and centrifuged again for 30 s at maximum speed. The analysis for FcRn and FCGR2 (1:100) was performed by Western blot, as described in Section 2.7.

### 2.9. Statistics

Data were assessed for statistical significance using unpaired t-test or Mann–Whitney test (GraphPad Prism 8, Version 8.3.0 (538), San Diego, CA, USA). The number of experimental repetitions is divided into technical replicates (*n*) and biological replicates (N). Biological replicates represent different batches of primary cells, different pigs or the number of newly cultured RPMI 2650 cells.

## 3. Results

So far, the transport pathways of IgGs through the nasal mucosa remain to be elucidated. Previous studies identified that FcRn appears to be involved in IgG trafficking [15]. Therefore, the focus of the present study is the interaction of IgGs with Fc receptors in models of the olfactory mucosa. In former studies, especially the glycosylation status of IgGs affects the binding to Fc receptors [31,41]. Several models have been developed so far to test the permeation of drugs through the olfactory epithelium with ex vivo tissue explants, primary nasal cells and tumor cell lines such as RPMI 2650 [13,36,39,42,43]. Here, the IgG permeation was evaluated in these models as well as the relevance of using allo- or xenogenic IgGs and their glycosylation.

### 3.1. IgG Permeation through Different Models of the Olfactory Mucosa and Olfactory Epithelium

#### 3.1.1. Impact of IgG Species Origin on Trans-Epithelial Permeation of IgGs

**Figure 2:**
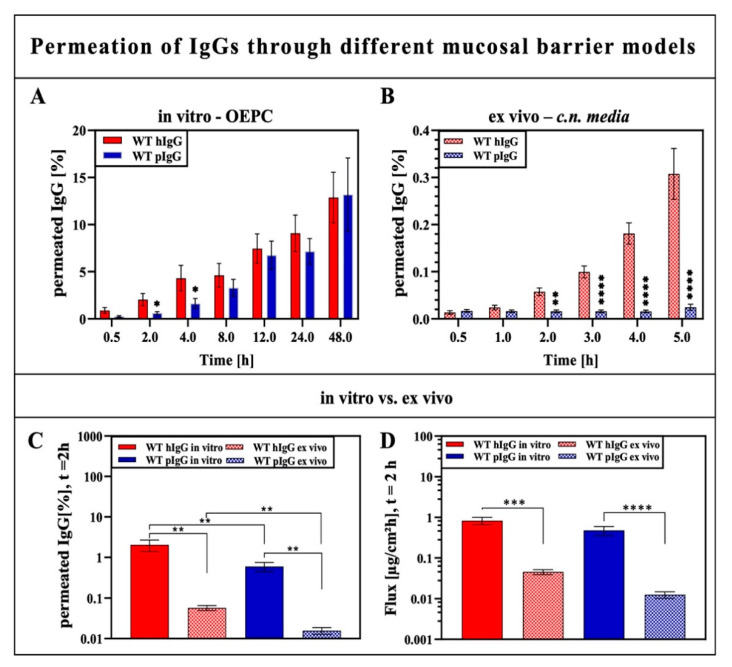
Cumulated permeation of allogenic porcine serum IgG (pIgG) and xenogenic human IgG (hIgG) through in vitro porcine olfactory epithelial primary cells (OEPC) and ex vivo porcine olfactory mucosa (*conchae nasalis media* (*c.n. media*)). (**A**) Percentage permeation of wild-type hIgG (WT hIgG) and porcine serum IgG (WT pIgG) through olfactory epithelial primary cells (OEPC) over 48 h. Error bars represent mean ± SEM. *N* = 4, *n* = 21. (**B**) Percentage permeation of WT hIgG and WT pIgG through excised nasal mucosa tissue explants (ex vivo—*c.n. media*) over 5 h. Error bars represent mean ± SEM. WT hIgG: *N* = 4, *n* = 29, WT pIgG: *N* = 4, *n* = 12. (**C**) Comparison of percentage permeated IgG through the OEPC in vitro model and the ex vivo model after 2 h. (**D**) Comparison of the flux of the IgGs through the in vitro and the ex vivo model. The significance was analyzed by unpaired t-test. * *p* < 0.05, ** *p* < 0.001, *** *p* < 0.0001; **** *p* < 0.00001, error bars represent mean ± SEM.

To evaluate whether there is a difference between the permeation of porcine serum IgGs (pIgG) and a human IgG (hIgG) through porcine nasal mucosa models, the permeation was determined using in vitro ALI cultures of OEPC and RPMI2650 as well as ex vivo tissue explants (Figure 2A–D). The results can give further information about the validity of the porcine model to develop nasal drug delivery with human antibodies since the human nasal olfactory mucosa is limited for such experimental models. All antibodies used here were normally glycosylated (wild types, WT).

Using OEPC ALI cultures derived from primary epithelial cells of the porcine olfactory mucosa cultured at the air–liquid barrier, the permeation of the hIgG was significantly faster over the first four hours in comparison to pIgGs. From 8 to 48 h, the permeation rate of pIgGs converges (Figure 2A). By contrast, using an ex vivo model derived from a specimen of the porcine olfactory mucosa, the hIgG permeation was 12 times higher after 5 h than the permeation of pIgG (Figure 2B). A comparison of the ex vivo and the primary cell in vitro model shows 35-fold higher permeation for hIgG and 1.8-fold higher flux in the in vitro model. For pIgGs, 35-fold higher flux and 37-fold higher permeation could be determined in the in vitro model compared to the ex vivo model. In brief, a difference in the permeation behavior of pIgG and hIgG was shown for the uptake at earlier time points in the epithelial layer model OEPC. This might indicate either different binding affinities to transport-related receptors or differences in the intracellular IgG trafficking. Furthermore, when using the ex vivo model of the *c.n. media*, pIgG can be hardly found at the basolateral/abluminal compartment in contrast to hIgG. Again, this suggests that species-dependent binding to IgG receptors or species-dependent IgG trafficking and/or degradation pathways influences the permeation of IgGs through the olfactory mucosa.

#### 3.1.2. Impact of IgG Glycosylation on Permeation through the Nasal Epithelium or Nasal Mucosa

The glycosylation of IgG was shown to have a high impact on Fc receptor binding in several studies [18,27,30]. However, the common opinion is that binding to the IgG transporter FcRn is not affected by deglycosylation of the IgG [44]. To evaluate the involvement of IgG glycosylation sensitive receptors in intranasal IgG permeation, a study was performed using a deglycosylated monoclonal hIgG (DG hIgG). The enzyme used for deglycosylation was EndoS, which hydrolyses the asparagine-linked glycans on IgG [41,45,46]. The experiment was not performed with polyclonal porcine serum IgGs, as a defined glycopattern was desired to simplify the analysis. The (de-)glycosylation of hIgG was evaluated by hydrophilic interaction liquid chromatography (HILIC, see Supplementary Figure S1).

**Figure 3:**
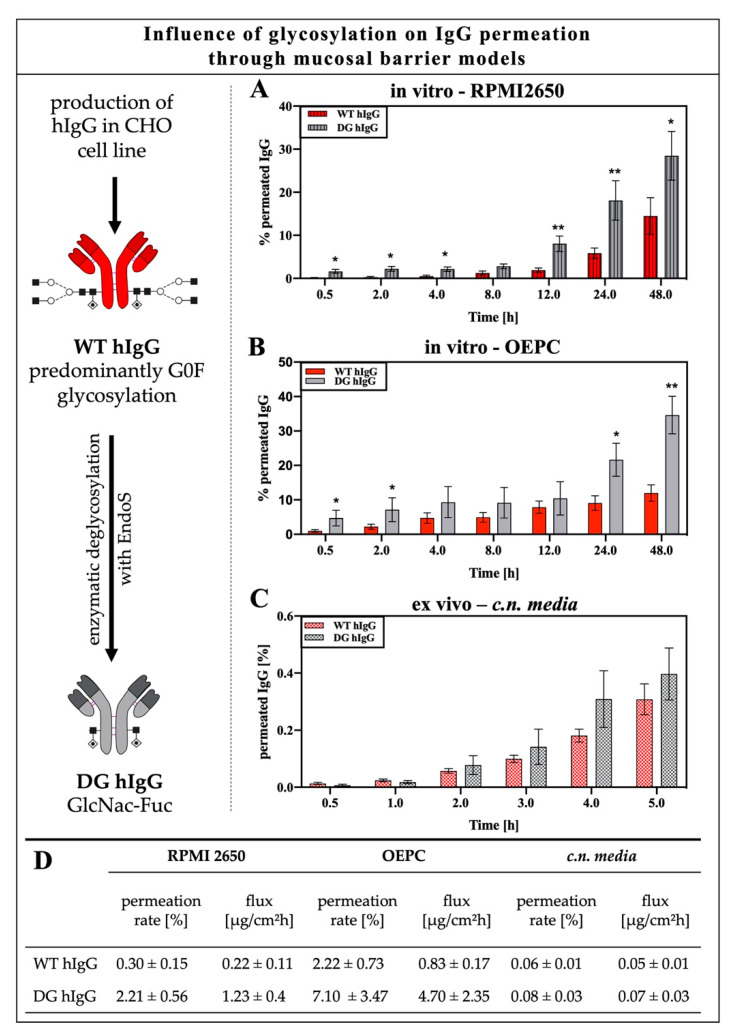
Permeation of wild-type (WT hIgG) and deglycosylated human IgG (DG hIgG) through RPMI 2650, porcine olfactory epithelial primary cells (OEPC) and porcine olfactory mucosa (c.n.media) (**A**) Percentage permeation of WT hIgG and DG hIgG through RPMI 2650 cells over 48 h. Error bars represent mean ± SEM. *N* = 3, *n* = 11. (**B**) Percentage permeation of WT hIgG and DG hIgG through olfactory epithelial primary cells (OEPC) in vitro model over 48 h. Error bars represent mean ± SEM. WT hIgG: *N* = 4, *n* = 21, DG hIgG: *N* = 3, *n* = 10. (**C**) Percentage permeation of WT hIgG and DG hIgG through excised nasal mucosa tissue explants (ex vivo) over 5 h. Error bars represent mean ± SEM. WT hIgG: *N* = 4, *n* = 29, DG hIgG: *N* = 3, *n* = 10. (**D**) Tabular summary of permeation rate and flux of WT and DG hIgG in the in vitro models RPMI 2650 and OEPC and the ex vivo model c.n media after 2 h. Results represent mean values ± SEM. Permeation of wild-type (WT hIgG) and Endo S digested deglycosylated human IgG (DG hIgG) through porcine olfactory epithelial primary cells and porcine olfactory mucosa. Both in vitro models show significant differences (*p* < 0.001) in permeation rate and flux compared to the ex vivo model. Furthermore, the permeation through the OEPC model resulted in significantly higher permeation and flux compared to the RPMI 2650 model (*p* < 0.001). The significance was calculated by unpaired t-test. * *p* < 0.05, ** *p* < 0.001, error bars represent mean ± SEM.

To avoid experimental bias due to cross-species binding, the permeation was performed in RPMI 2650 with allogenic human IgGs in addition to the permeation through OEPC and *c.n. media*. The cell line RPMI 2650 was derived from cancerous human nasal squamous epithelium and is currently used as a standard model for intranasal drug delivery since specimens of human nasal mucosa are unfortunately hardly available [47,48,49]. Comparable to the in vitro permeation through OEPC layers (Figure 3B), DG hIgG showed a higher permeation rate and flux than wild-type IgG in the RPMI 2650 ALI model (Figure 3A). In detail, the percentage permeation of DG hIgG was two times higher compared with the WT hIgG. The permeation of DG hIgG and WT hIgG through OEPC resulted in three times higher permeation of the DG hIgG after 48 h (Figure 3B). By contrast, the permeation rate of DG hIgG and WT hIgG did not show significant differences in the ex vivo model but a tendency towards higher permeation was observed for DG hIgG (Figure 3C). Considering the direct comparison of the permeation results after two hours, the permeation of WT hIgG through the primary cell in vitro model was 40 times higher than the permeation through the tissue explant (Figure 3D). For DG hIgG, the percentage in vitro permeation was over 80 times higher than the ex vivo permeation after 2 h (Figure 3D). Compared to the ex vivo permeation, both variants of the IgG showed a higher permeation rate and higher flux in the RPMI 2650 model at 2 h. In contrast, the permeation and the flux through the primary cell model was significantly higher than the permeation through RPMI 2650 cells for both the DG hIgG and the WT hIgG. This was surprising as the barrier tightness of RPMI 2650 (transepithelial resistance: 50–70 Ωcm^2^) is much lower than OEPC (transepithelial resistance: 800–1000 Ωcm^2^) and therefore the RPMI 2650 was suspected to show higher IgG flux due to paracellular transport [36].

In contrast to the interspecies differences in permeation from Figure 2, the permeation behavior of DG und WT hIgG displayed similar patterns in the in vitro (OEPC, RPMI 2650) and ex vivo (*c.n. media*) models. In all in vitro models, deglycosylation of hIgG did result in higher permeation of hIgG from the apical to the basolateral side. Moreover, in the ex vivo model, the tendency of higher permeation of DG hIgG is visible but not significant due to the usually high standard variations in this model type. The higher permeation rate of DG IgG could indicate a glycosensitive mechanism in IgG trafficking. Receptors which are known to be sensitive to Fc glycosylation are FCGRs. The significantly higher permeation of DG hIgG could imply the involvement of FCGRs either in IgG recycling or degradation.

### 3.2. Analysis of IgG Transporter in Porcine Nasal Mucosa, Primary Epithilial Cells and the RPMI 2650 Model

#### 3.2.1. FcRn Expression in OEPC and RPMI 2650

Up to now, FcRn is the best described protein, which has an important role in IgG transport. Further, enhanced transport via FcRn was shown by Fc engineering [16,50]. We recently described the expression of FcRn in porcine nasal mucosa explants [13]. Therefore, we now focused on the primary cell model (OEPC) and the human nasal cell line RPMI 2650.

**Figure 4:**
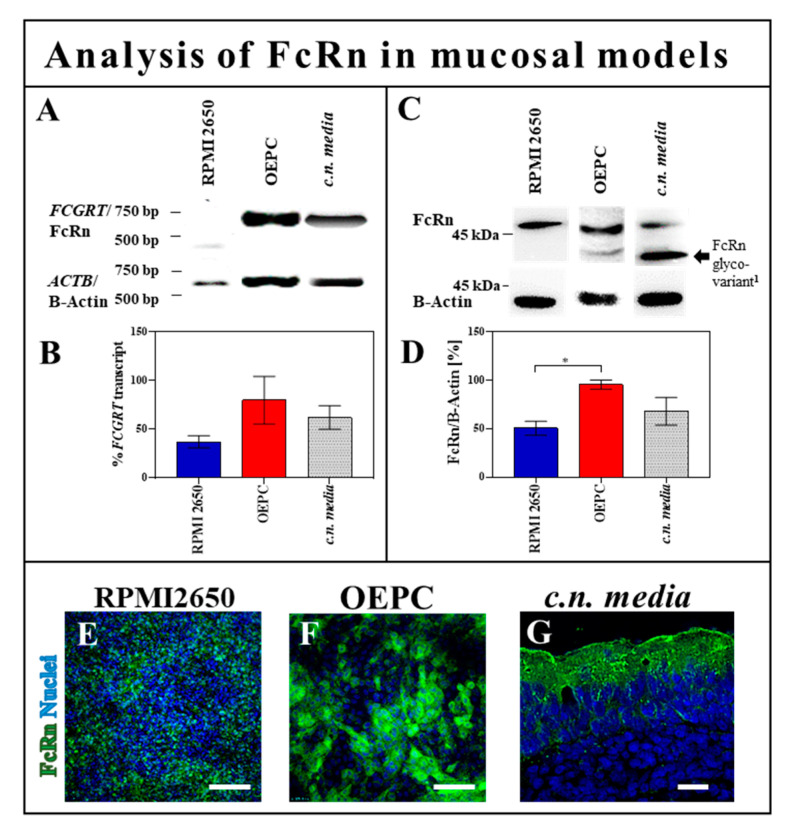
FcRn expression in olfactory epithelial primary cells and RPMI 2650 cells. (**A**) Reverse transcription PCR: *FCGRT* (gene of FcRn) transcription analysis in porcine olfactory epithelial primary cells (OEPC; expected size of amplified cDNA: *FCGRT* 635 bp, *ACTB* (gene of B-Actin): 612 bp), the human cell line RPMI 2650 (expected band size: *FCGRT*: 403 bp, *ACTB*: 612 bp) and the reference tissue from porcine olfactory mucosa (*c.n. media*). (**B**) A relative quantification was carried out by comparing the band intensity with a housekeeping gene. The significance was calculated by comparison of the OEPC and RPMI 2650 data with the *c.n. media* transcription data using an unpaired t-test. *n* = 4; error bars represent mean ± SEM. (**C**) Western blot: FcRn expression analysis in OEPC, RPMI 2650 and *c.n. media* (B-Actin: 42 kDa., human and porcine FcRn: 42 kDa–46 kDa **^1^** depending on glycovariant) (**D**) For the quantification of multiple bands due to different glycovariants of the FcRn [14], intensities were summed up. Statistical analysis was performed as described for the transcription analysis using an unpaired t-test. * *p* < 0.05, *n* = 4; error bars represent mean ± SEM. (**E**) Immunofluorescence study of FcRn expression (green) in RPMI 2650, OEPC (**F**) and *c.n.media* (**G**). Differences in cell morphology and size of OEPC and RPMI 2650 were discussed recently [36]. Scale bar: 50 µm.

The transcription (Figure 4A,B) and the expression of *FCGRT* (gene of FcRn) in OEPC and RPMI 2650 (Figure 4C,D) were determined by RT PCR and Western blot and compared to porcine olfactory mucosa (*c.n. media*) as a reference [13]. There was a tendency observable for a lower expression level of FcRn in RPMI 2650 in comparison to OEPC and porcine *c.n. media* tissue, but this failed to reach statistical significance. FcRn expression in OEPC was comparable to *c.n. media* as the reference tissue. In both models, the intensity of FcRn immunofluorescence (Figure 4E,F) was roughly comparable with the semi-quantification by Western blot. It should be noted that different secondary antibodies had to be used to detect human and porcine FcRn and should hence not be compared head-to-head (Figure 4C–G). The differences in the cell morphology and size of RPMI 2650 and OEPC were described in detail recently [36]. In summary, FcRn expression was found in each of the three models even though differences in the expression levels were observed.

#### 3.2.2. Protein Interaction Study of FcRn and FCGR2 with Wild-Type and Deglycosylated IgG

It was previously demonstrated that the affinity of FcRn to IgGs with G0F-gycosylation is not significantly altered to Endo S digested (deglycosylated) IgGs deglycosylation, leaving a GlcNac-Fuc glycosylation pattern, as was the case for DG IgG in this study [51]. Hence, the significantly higher levels of DG IgG compared to WT IgG in both epithelial models (Figure 3A,B) appear to be linked to a glycosylation dependent Fc-interacting molecule, which allows faster and more efficient transport of DG IgG through the epithelial layer. Further, we may exclude that nasal epithelial IgG trafficking is only dependent on FcRn because of the insensitivity of FcRn to alteration of Fc-glycosylation [52]. To narrow down the number of potential glycosylation-sensitive Fc-interaction molecules, we focused here on FCGR, since their dependence on glycosylation was well demonstrated in many studies. Table 2 details and justifies why a particular focus was placed on FCGR2. Briefly, FCGR subclasses have a different level of sensitivity to Fc-glycosylation patterns. While FCGR1 is largely independent of Fc glycosylation, especially FCGR3a shows high sensitivity to fucose residues. Even when the Fc was trimmed back by Endo S to a GlcNAc-Fuc residue, a 10-fold reduction in affinity was reported [53]. In general, FCGR2 and FCGR3a show almost a complete loss of affinity towards deglycosylated IgG [29].

As FCGR1 is insensitive to the Fc-glycopattern and FCGR3a is highly fucose sensitive, we assumed that the involvement of these receptors would not yield a remarkable difference in the permeation pattern of the fucosylated hIgG WT and the still fucosylated hIgG DG (GlcNAc-Fuc) [29,53]. With this conclusion and due to the link between FCGR2 and IgG trafficking observed before in placenta, we focused here on FCGR2 [26].

**Figure 5:**
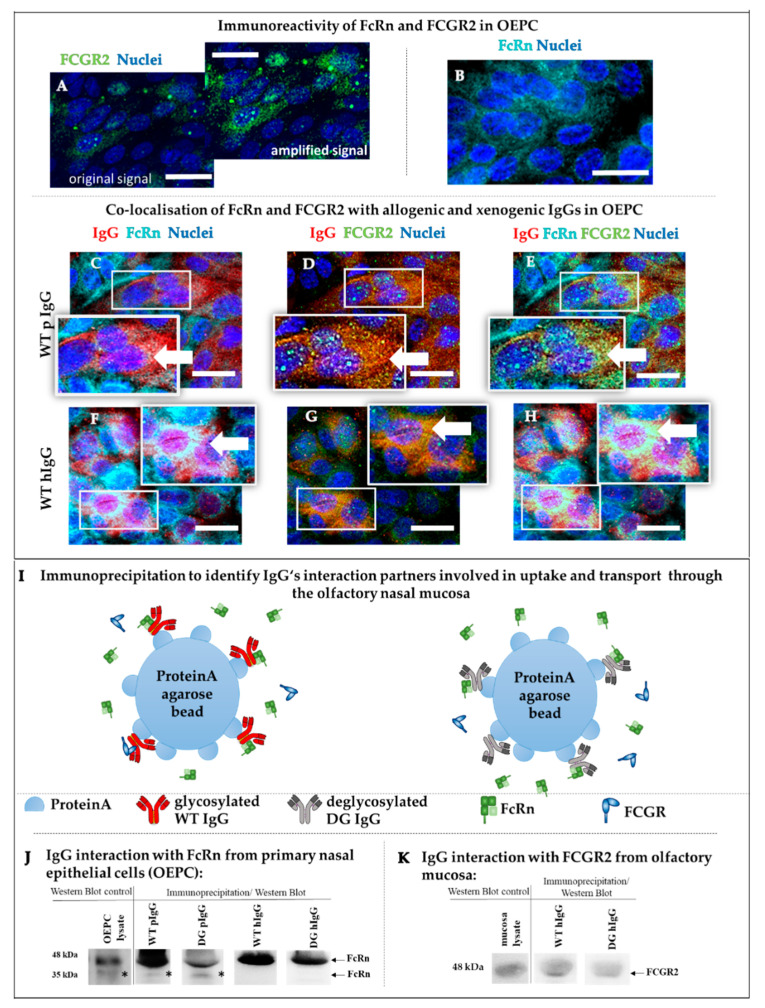
Analysis of IgG transporter expression and protein–protein interaction. (**A**–**H**) Co-localization study of FcRn (**B**,**C**,**F**) and FCGR2 (**A**,**D**,**G**) with porcine (**D**,**E**) and human (**F**–**H**) IgG in primary cells from olfactory epithelium (OEPC) (representative data are shown, *N* = 3, *n* = 3). (**A**) To visualize the low amount of FCGR2 (original), the intensity was highly increased (increased intensity) to evaluate co-localization with FcRn and IgG. FCGR2 expression was found to be mainly intracellular. (**B**) FcRn was found to be intracellular and on the cell’s surface with sufficient immunoreactivity. (**C**–**E**) Co-localization of FCGR2 (green), FcRn (cyan) and pIgG (red). (**F**–**H**) Co-localization of FCGR2 (green), FcRn (cyan) and hIgG (red). Scale bar: 20 µm, arrows mark sites of co-localization. (**I**) Underlying principle of the immunoprecipitation (IP) to evaluate the binding of wild-type (WT hIgG) and deglycosylated human IgG (DG hIgG) and porcine IgG (WT pIgG, DG pIgG) to the porcine FcRn. (**J**) IP study of FcRn interaction with wild-type and deglycosylated allogenic pIgG and xenogenic hIgG using primary cell lysate (OEPC, origin: porcine nasal mucosa, olfactory region) (*N* = 3). IgGs were incubated with OEPC cell lysate and captured using Protein A agarose beads. The resulting bands could not be quantified due to high batch-to-batch variations in the cell lysates. * The additional band in the line of the WT and DG pIgG is caused by different glycovariants of the porcine FcRn [14]. hIgGs seem to bind only to one of the variants. (**K**) Interaction study (IP) of WT hIgG and DG hIgG with FCGR2 using whole mucosa lysate from *c.n. media*.

FCGR2-immunoreactivity is rather low in comparison to FcRn (Figure 5A,B); therefore, the signal intensity was amplified using Image J software (Figure 5A) in order to visualize potential co-localization of FCGR2 with IgG. Nevertheless, co-localization of FcRn and FCGR2 could be shown in OEPC in permeation experiments of pIgG (Figure 5E) and hIgG (Figure 5H). Unfortunately, for pigs, only an antibody against both variants of the FCGR2 is commercially available and therefore the results cannot distinguish between FCGR2a and b variants. By RT-PCR, we could detect FCGR2b in *c.n. media* (data not shown). Interestingly, not all epithelial cells that appeared to have taken up IgG showed immunoreactivity against one or both of the Fc receptors (Figure 5E,H).

To investigate the interaction between allogenic pIgG and xenogenic hIgG with porcine FcRn, immunoprecipitation was performed (Figure 5I–K). IgGs were bound to Protein A agarose beads, and a pull down with lysates from OEPC containing endogenous levels of FcRn was performed. To yield enough cell lysate for the experiment, each analyte IgG was incubated with OEPC cell lysate from different batches. We found protein interaction of porcine and human IgG in their wild-type form as well as in their deglycosylated form with the FcRn (Figure 5J). The analysis resulted in two bands because of the different glycopatterns of the FcRn, as described above [14]. However, the human IgGs did not show the lower band, indicating that human IgGs bind only to one of the two glycovariants. Differences in band intensity were inconsistent between repetitive experiments. Therefore, no quantification was performed. Most probably, the batch-to-batch variability of the primary cell composition and cell count used for the cell lysates causes these problems which we have not solved so far. This heterogeneity of OEPC cell lysates was described in more detail before [36]. However, the interaction of the WT and the DG variants for pIgG and hIgG with FcRn could be shown in OEPC cell lysates.

Due to the co-localization observed in Figure 5H, we performed immunoprecipitation also for interaction with FCGR2. However, immunoprecipitation for interaction of IgG and FCGR2 failed in OEPC cell lysates (data not shown), while a detection was possible in lysates of *c.n. media* whole tissue explants (Figure 5K). This could indicate a problem with the detection limit as FCGR2 is low expressed in epithelial cells (see Figure 5A). Unfortunately, there are no studies reported that have previously compared FcRn and FCGR expression head-to-head in pigs. Indeed, there is evidence that the expression level of FCGR2 is directly linked to cytokine expression in the organism [57]. As slaughterhouse pigs were used in this study, we cannot exclude experimental bias due to the health state of the pig. Furthermore, the choice and availability of antibodies against porcine FCGR is rather limited and, hence, no other antibodies against these antigens could be tested. Therefore, the interaction signals might be below detection limit of the Western blot.

## 4. Discussion

The understanding of IgG transport processes from the nasal mucosa to the brain is an important step on the way to developing specialized, intranasally applied, therapeutic IgGs against CNS-related and oncologic diseases. Many studies have used different approaches to investigate the general transport processes of antibodies in mucous membranes. Most of these studies concluded that FcRn plays a key role in antibody transport [58,59,60,61,62]. Hereby, apical monomeric IgGs are taken up via pinocytosis and are bound intracellularly by the FcRn under slightly acidic conditions in early endosomes [15]. Afterwards, the IgGs are recycled, as is known for endothelial cells, or transcytosed to the lamina propria in polarized epithelial cells [63].

In this study, first, the involvement of FcRn in IgG transport through porcine olfactory mucosa was evaluated in vitro via OEPC ALI culture models and ex vivo models. According to common knowledge, the FcRn is the main transporter in terms of IgG trafficking in epithelial and endothelial barriers. However, there are still open questions concerning IgG trafficking, especially from the nasal mucosa to the brain [23,25,64]. To simplify the complex mucosa with the highly heterogeneous epithelial layer containing gland cells, ciliated cells, immune-related cells, the mucous layer and the underlying lamina propria including blood vessels, glands and the local immune system, permeation experiments were performed in an epithelial primary cell ALI culture model to display only the first barrier of the mucosa. According to the literature, mucosal epithelial cells express the FcRn and are able to perform transcytosis to the underlying tissue [17,65]. The binding of IgGs to FcRn shows high cross-species reactivity; however, the binding affinities differ depending on the species [66,67,68]. It is known that the binding affinity and the FcRn-related transport is fairly similar for hIgG and pIgG in porcine epithelial cells. However, Stirling et al. showed that hIgGs are taken up more rapidly by porcine FcRn [14]. To gain further information about IgG transport in the epithelial barrier of porcine olfactory mucosa, the permeation rate and flux of hIgG and pIgGs were compared. The results shown in Figure 2A,B support the findings of faster uptake of hIgG. In early time points, the permeation rate as well as the flux of hIgG was higher compared to the pIgG but both assimilated after 8 h. We concluded in general that the permeation in the first epithelial barrier is not strongly dependent on IgG species origin. Nevertheless, by immunoprecipitation and immunofluorescence, we are the first to show an interaction of FcRn and IgG in the olfactory epithelial barrier roughly similar to other mucosal sites [14,15,69]. It must be mentioned that we used porcine serum IgGs and therefore cannot exclude IgG subclass-specific effects in our experiment as FcRn binding affinity depends on the IgG subclass [55]. However, there are still open questions in terms of the transcytosis process of IgG. It is, for example, unknown how the intracellular sorting of IgG is proceeded and how monoclonal IgG and immune complexes are distinguished [55]. In this context, also the pinocytosis uptake of IgGs in the epithelial cells can be addressed, as there is an acidic environment on the apical surface of the mucosal epithelium. Therefore, FcRn-mediated uptake would be possible as the binding affinity for IgG is highest around pH 6 to 6.5, which matches the pH of the mucous layer [70]. In contrast, Hornby et al. found evidence in 2014 for low FcRn surface expression in Caco-2 cells and a surface FcRn-IgG binding that was inconsequential for overall transcytosis [71]. In addition, immunofluorescence staining against FcRn in OEPC shows that FcRn is not expressed in all epithelial cells but varied heavily in the cellular monolayer, indicating that FcRn expression is highly dependent on the cell type [72]. Again, this supports the common assumption of an uptake based mainly on pinocytosis. However, there is evidence for a bidirectional transport of IgG mediated by FcRn in the MDCK cell line expressing human FcRn [63]. If there is FcRn-mediated IgG transport from the basolateral to the acidic apical side in the nasal epithelial layer, how does the release of the IgG take place? Again, the involvement of an additional mechanism including a factor X receptor protein might be possible, as postulated before by Sockolosky and Szoka in 2016 for the IgG recycling pathway [52].

Interestingly, in the complex ex vivo model, the permeation rate varies widely between the porcine and the human IgGs. Flux and permeation rates of the human IgG were significantly higher after 1 h in comparison to porcine IgG. This result is in accordance with a former immunofluorescence study in porcine olfactory mucosa and apparent degradation in lymphoid follicles [13]. Our data support the thesis of porcine IgG remaining in the tissue as the amount of porcine IgG recovered from the basolateral compartment was below or near to the detection limit and did not increase during the course of the experiment. In the former study, it was suggested that the species origin of the IgG is highly important for IgG transport and clearance in the nasal olfactory mucosa [13]. The data gained in the present study further indicate species-dependent IgG trafficking—however, not in the epithelial layer but in cellular structures of the underlying lamina propria. If FcRn would be the only transporter involved in IgG trafficking at the olfactory mucosa, it should be species-independent (regarding human and porcine IgG), as proven here in the in vitro model and by Stirling et al. in 2005 in tumor cells transfected with porcine FcRn [14]. Here, again, such a co-factor X involved in IgG trafficking could be considered.

In general, the permeation rate in the epithelial barrier model (OEPC ALI culture) was more than 35-fold higher for both IgGs compared to the ex vivo mucosa model after 2 h. Hereby, influencing factors such as the longer distance through the whole tissue, the connective underlying tissue as well as recycling and clearance at blood vessels or glands will reduce the amount of permeated IgG. However, the big difference between the human and the porcine IgG cannot be explained by these parameters if FcRn is assumed to be the only transporter involved. Instead, the missing link might be the mucosal immune system. The immune system in the porcine and human nasal mucosa is organized in so-called lymphoid follicles (LF) [13,73,74,75]. These LFs consist of B and T cell agglomerates as well as isolated monocytes and dendritic cells (DCs) [74,75]. Furthermore, beneath the epithelial layer, DCs and macrophages are located and involved in immune surveillance at the mucosal barrier [75,76,77]. We have shown recently that LF areas are spared from permeated IgG, indicating immune-related uptake and degradation processes [13]. Further, it is known from intestinal mucosal sites that transcytosed immune complexes are taken up by DCs directly beneath the epithelial layer. These immune complexes are taken up by other Fc binding receptors: the FCGRs. FCGRs are a heterogeneous group of cell surface glycoproteins that interact with antibody–antigen complexes and initiate defending responses in effector cells of the immune system. FCGRs regulate a variety of immune responses such as phagocytosis, degranulation, antibody-dependent cellular cytotoxicity, transcriptional regulation of cytokine and chemokine expression, B cell activation and immune complex clearance [78]. A hint of a direct or indirect interaction of FcRn with FCGRs is the FcRn-mediated intracellular routing to cross-presentation compartments in DCs [55]. However, the exact intracellular mechanism responsible for directing immune complexes remains largely unclear.

**Figure 6:**
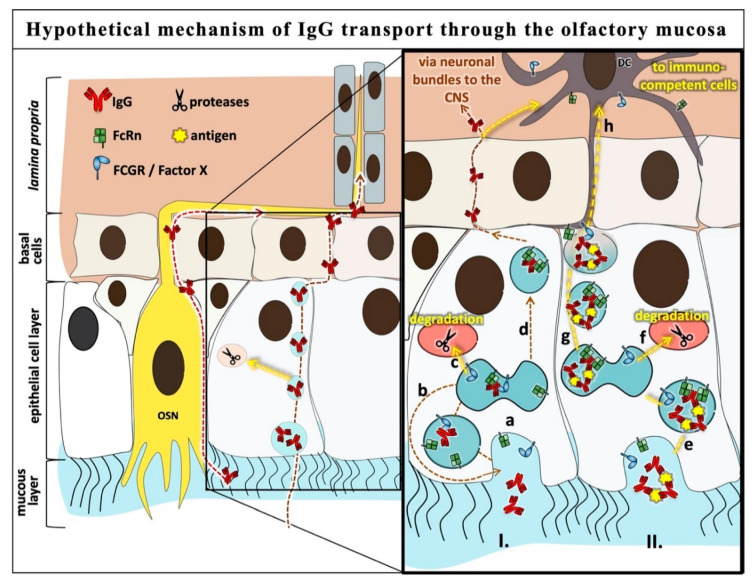
Cross-binding hypothesis: hypothetical mechanism of IgG transport through the olfactory mucosa. I. (a) Uptake of monomeric IgGs was presumably via pinocytosis, but FcRn was also found at the apical surface [13] and vesicle transport to the sorting compartment. (b) Cross-binding of IgG with FcRn and a second Fc receptor such as FCGR2, which might be factor X as suggested [52]; recycling back to the apical surface or (c) lysosomal degradation. Alternatively, a second FcRn replaces FCGR2/factor X and (d) the monomeric IgG is transcytosed to the *lamina propria*/basolateral side. II. (e) Uptake of immune complexes similar to monomeric IgG and vesicle transport to the sorting compartment. Binding to FCGR2/factor X is not possible due to steric hindrance; therefore, double binding of FcRn to one Fc part is possible. The complex can either (f) be degraded in the lysosome or (g) transcytosed to the *lamina propria* and (h) taken up by dendritic cells (DC) via different FCGRs or FcRn. Further studies are needed to confirm the role of FCGR2 in IgG trafficking in the airway epithelia. Due to limitations of the commercially available anti-porcine FCGR2 antibodies, we could not distinguish between FCGR2a and FCGR2b. Nevertheless, other studies suggest FCGR2b to be expressed in epithelial cells.

Are FCGRs the missing factor X and are they involved in IgG trafficking in the epithelia? In 2019, Golebski et al. published the involvement of FCGR3 and TLR4 in discrimination between commensal and invading bacteria in human nasal epithelial cells [56]. The expression of FCGR in the nasal epithelium strengthens the hypothesis of crosstalk of FCGR and FcRn in IgG transport and sorting processes in the epithelium. Another interesting study was carried out by Ishikawa et al. in 2015 that demonstrated maternal IgG trafficking in human placental endothelial cells mediated by FCGR2b in the absence of FcRn [26]. They further suggest that IgG internalization and transcytosis is mediated rather by FCGR2b than FcRn in the placental endothelium. This could explain why serum albumin, which is also recycled by FcRn, is not transcytosed across the placental barrier [79]. Hence, based on the expression of FCGR2 in OEPC, FCGR2 could be a possible candidate in the search for factor X (see Figure 6). However, species-related specials have to be considered as, for example, unlike in humans, maternal IgGs do not cross the porcine placenta to reach the fetus, even though FcRn is expressed there [80].

The binding of IgG to low-affinity FCGRs is highly dependent on the glycosylation of the IgG despite the binding to the high-affinity receptor FCGR1 [53]. To directly gain some ideas regarding which FCGR type might be involved in nasal IgG transport, a fucosylated human IgG1 was deglycosylated using the enzyme Endo S that leaves a glycan residue of N-Acetylglucosamine (GlcNac) and fucose at asparagine 297 (see Supplemental Figure S1; for details concerning the Fc-glycosylation sensitivity of different FCGR types, see Table 2). This IgG and its glycosylation pattern were described previously [41,46]. It should be noted that a human IgG was used due to the lack of commercially available monomeric porcine IgGs with a defined glycopattern. However, a recent study showed comparable binding of human and porcine IgG to the porcine FCGR1, FCGR2a and FCGR2b but no interaction with the porcine FCGR3a [35]. Therefore, we cannot make any assumptions about the involvement of this receptor type. However, as the human IgG used in this study is fucosylated (GalNac-Fuc) and the common IgG fucosylation patterns are known to highly decrease the affinity to FCGR3a, an interaction is rather unlikely [53]. If the high-affinity FCGR1 would be the only FCGR involved in nasal IgG trafficking, no difference between the WT IgG and the DG IgG should be detected as it is known to be mainly unaffected by deglycosylation in human. Hence, since FCGR2b was the only FCGR described to be involved in epithelial IgG trafficking we focused on this subclass.

Significantly higher levels of DG IgG were detected in the epithelial models compared to WT IgG (Figure 3). In the ex vivo nasal mucosa model, a tendency towards higher levels was detected; however, due to the high standard variation, this failed to demonstrate statistical significance. It was demonstrated that the affinity of FcRn to IgG is rather unaffected by deglycosylation of IgG; hence, we can exclude the notion that nasal epithelial IgG trafficking is only dependent on FcRn [51,52]. Further, the higher flux of DG IgG in the ex vivo mucosa model may indicate that other Fc receptors like FCGRs are not only involved in IgG trafficking through the epithelial layer but also in crosstalk to cells of the *lamina propria* (Figure 6).

To exclude interspecies effects due to the use of human IgG in porcine tissue and cells, the permeation experiments were repeated using the nasal mucosa standard cell line RPMI 2650 [37,39,49]. The results from the OEPC model could be confirmed in the RPMI 2650 model, indicating again a cross-species mechanism for IgG trafficking. The permeation rate in RPMI 2650 was unexpectedly low compared to the permeation rate through the primary cell layer. It was shown before that OEPC forms a much tighter barrier compared to the RPMI 2650 cells [34]. Furthermore, RPMI 2650 cells grow in multilayers and do not completely polarize like primary epithelial cells [36,37,81]. Apparently, the amount of FcRn directly correlates with IgG flux even in this rather leaky model. OEPC demonstrated the highest protein expression followed by RPMI 2650 cells and olfactory mucosa (Figure 4). In an immunoprecipitation study using OEPC lysates, little tendency of lower FcRn interaction when using deglycosylated IgG could be shown. However, due to high variabilities between the samples with cells of different batches, it cannot be considered as a significant result. Nevertheless, a clear interaction between IgG and FcRn was shown. The correlation between IgG uptake and FcRn expression, together with the results from the immunoprecipitation study and the permeation study using DG IgG, suggest that it is likely that FcRn is involved in IgG trafficking in the nasal mucosa, but not exclusively.

Since deglycosylated IgGs show higher permeation rates in all models, there was the assumption of an IgG glycosylation sensitive factor X that is involved in the transport. As FCGR2 was already identified to be involved in IgG trafficking in the placenta, this target was the first attempt to identify the factor X [26].

So far, identification of FCGR2 transcripts has yet not given any reliable results, possibly due to the lack of valid mRNA sequences for porcine FCGR transcripts. Therefore, an immunofluorescence study was performed to check for FCGR2 expression. Unfortunately, we cannot distinguish between the subtypes a, b and c of FCGR2 in this study. Nevertheless, co-localization was found between FcRn, IgG and FCGR2 for both IgGs in the OEPC model (Figure 5C–H). Without interaction studies of FcRn together with FCGR2b and IgG, a final conclusion cannot be made. As described above, also FCGR3a was found in nasal epithelial cells. However, we can exclude this receptor from being involved in this study as we used deglycosylated hIgGs that still contain a fucose residue in their glycosylation pattern. As mentioned before, even a small glycosylation residue containing fucose will significantly reduce interaction with FCGR3 and therefore the permeation pattern in this study should not have changed between hIgG WT and hIgG DG [82]. There are several facts in favor for the hypothesis of FCGR involvement. For example, an IgG3 variant with hinge-region deletions that prevent binding to FCGR but not to FcRn was still transported across the human placenta [83]. Likewise, deglycosylated IgGs were transported in mice [84]. Furthermore, a comparison of glycosylation patterns between fetal and maternal IgG by Stapelton et al. in 2018 concluded that IgG transport was not glycosylation selective [85]. Regardless, the differences in permeation rate between WT and DG IgG, together with the differential transport of IgG subclasses described in several studies, suggests that there is at least one factor X involved in IgG transport in the nasal mucosa in addition to FcRn [61,86,87].

## 5. Conclusions

In the present study, we showed, for the first time, the differences in permeation rate between allogenic and xenogenic IgGs as well as WT IgG and DG IgG in ex vivo olfactory mucosa, primary olfactory epithelial cells and RPMI 2650.

In this study, we present new insights into IgG trafficking. On the one hand, we showed that permeation with IgG from alien species did not result in an altered permeation rate in the epithelial barrier but in the underlying lamina propria. In more detail, the permeation rate of pIgG and hIgG did not differ in the epithelial barrier model but in the whole tissue explant. Hereby, as seen in immunofluorescence studies before, pIgG crosses the mucosa in very low amounts or not at all, whereas hIgG was detectable in higher levels at the basolateral side [13]. Most probably, this effect could be explained by the immune-related uptake of the pIgGs and the different binding affinities to the porcine FCGR2 and FCGR3 [35]. On the other hand, we show strong evidence for the dependence of IgG transport on the glycosylation status of the IgG.

DG IgG showed significantly higher permeation rates in all models compared to the WT IgG. The highest difference was shown in the primary cell model and therefore in the first epithelial layer of the mucosa. These results could hint at an additional mechanism including a still unknown factor X involved in IgG trafficking in epithelial cells that is dependent on the IgG glycosylation. In Figure 6, the cross-binding hypothesis that is derived from the data of this study is displayed.

The underlying principle of the cross-binding hypothesis is the idea that antibody agglomerates that have bound to an antigen, so-called immune complexes, are transferred to immune competent cells such as DCs in the lamina propria. Such a mechanism would ensure that the mucosal surface is monitored by the underlying immune system to prevent infection. In contrast, the transcytosis of unbound, monomeric IgGs from the apical to the basolateral side may not be very important for immune surveillance. Therefore, it would make sense that it may occur as an occasional event or due to a high antibody concentration on the apical surface as it can occur during intranasal delivery. It may also be possible that the suggested factor X is expressed in a substantially lower amount compared to FcRn and therefore a double binding of Fc by two FcRn proteins is enabled if a high amount of IgG is present on the apical side of the epithelial cell. To distinguish between monomeric IgG and multimeric immune complexes, the additional factor X might be involved. In this hypothesis, factor X binds to the glycosylated hinge region of IgG in combination to FcRn to ensure either lysosomal IgG degradation or recycling back to the apical surface (Figure 6I). In immune complexes, the hinge region might not be freely accessible and may not bind to factor X due to steric hindrance. In this case, double binding or multiple binding of FcRn could be possible and might give the signal for transcytosis. The basolateral endocytosed immune complex could then be taken up by DCs via FCGRs (Figure 6II).

Due to the fact that, for most FCGRs, their affinity to IgG Fc is dependent on Fc glycosylation, it is likely that the suggested factor X could be one of the FCGRs or a not yet identified Fc binding protein. Because of literature based data, we made the first attempt to verify the involvement of FCGR2 not only in the placenta but also in the olfactory epithelium [26]. However, further research is needed to confirm these observations and the use of this transcytosis mechanism for IgG drug delivery in the airway.

## Figures and Tables

**Table 1:** List of secondary antibodies used in this study.

Antibody	Antigen	Host	Source, Catalog Number
Anti-rabbit IgG-Rhodamine Red^TM^-X	Whole molecule rabbit IgG	Donkey	Jackson Immuno Research Europe Ltd., Cambridgeshire, UK, Cat. #711-295-152
Anti-murine IgG-Alexa Fluor^®^ 488	Whole molecule mouse IgG	Goat	Jackson Immuno Research Europe Ltd., Ely, UK, Cat. #115-545-003
AffiniPure Anti swine IgG (H+L)-Alexa Fluor^®^ 647	Whole molecule porcine IgG	Goat	Jackson Immuno Research Europe Ltd., Ely, UK, Cat. #114-605-003
AffiniPure Anti human-Alexa Fluor^®^ 647	Whole molecule human IgG	Donkey	Jackson Immuno Research Europe Ltd., Ely, UK, Cat. #709-605-149
Anti-rabbit IgG-HRP	Whole molecule rabbit IgG	Goat	Jackson Immuno Research Europe Ltd., Ely, UK, Cat. #111-035-003
Anti-murine IgG-HRP	Whole molecule rabbit IgG	Goat	Sigma Aldrich, Taufkirchen, Germany, Cat. #AP5278
Anti-human κ light chain IgG-HRP	Human κ light chain	Goat	SouthernBiotech, Birmingham, AL, USA, Cat. #2060-05
AffiniPure Anti swine IgG (H+L)-HRP	Whole molecule porcine IgG	Goat	Jackson Immuno Research Europe Ltd., Ely, UK, Cat. #114-035-003
AffiniPure Anti swine IgG (H+L)	Whole molecule porcine IgG	Goat	Jackson Immuno Research Europe Ltd., Ely, UK, Cat. #114-005-003

**Table 2:** Fc receptors and their potential relevance in epithelial IgG trafficking.

Fc Receptor	Sensitivity to Deglycosylated IgG	Potential Relevance in Epithelial IgG Trafficking	Refs.
FCGR1	Rather insensitive due alternative binding domain.	Has not yet been implicated in IgG trafficking; expression in epithelial has not yet been demonstrated.	[30,32,53]
FCGR2	Endo S treatment of IgG decreases their interaction with FCGR2, but fucose does not alter the affinity to FCGR2.	Expression in epithelial cells from placenta was shown in several studies. Was linked to IgG trafficking of maternal IgG to the fetus in placental cell lines (FCGR2b).	[26,29,32,53,54]
FCGR3a	Endo S treatment abolishes the interaction with FCGR3, but also fucosylated IgGs such as hIgG used here were shown to bind with significantly decreased affinity.	Expression in human nasal epithelium was demonstrated. Controversially discussed for placental IgG trafficking.	[32,53,55,56]

## Supplementary Materials

The following are available online at https://www.mdpi.com/1999-4923/12/11/1014/s1, Figure S1: HILIC analysis of hIgG glycan pattern digested either in total (PNGaseF) or by Endo S.
