# Supplementary figures and images for: Impact of Glycosylation and Species Origin on the Uptake and Permeation of IgGs through the Nasal Airway Mucosa
Source: Pharmaceutics. 2020 Oct 23;12(11):1014. doi: 10.3390/pharmaceutics12111014 (PMC7690786; doi:10.3390/pharmaceutics12111014)

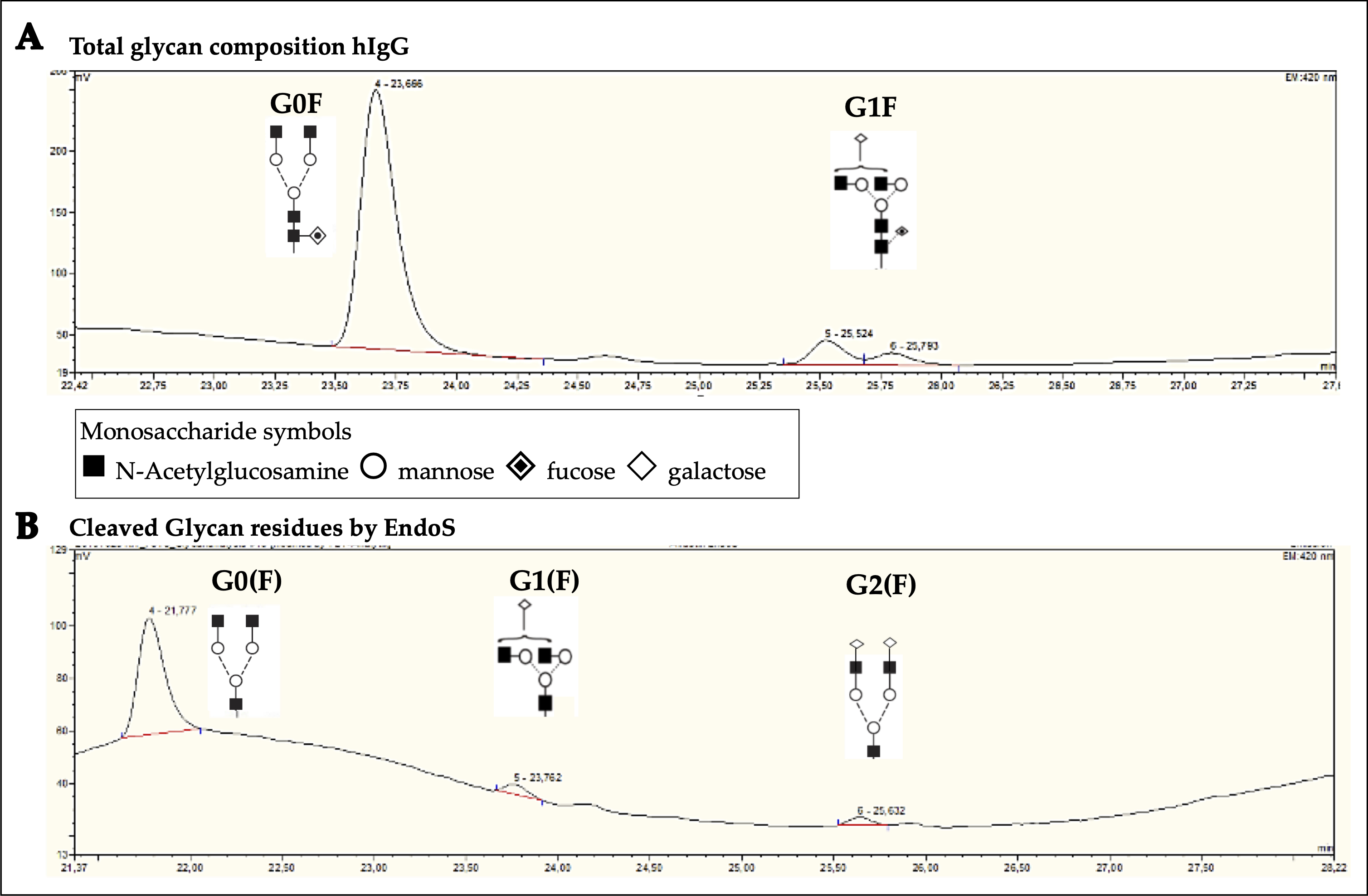

Supplement: Supplementary file 1 [file pharmaceutics-12-01014-s001.zip › pharmaceutics-962154-supplementary.jpg]
